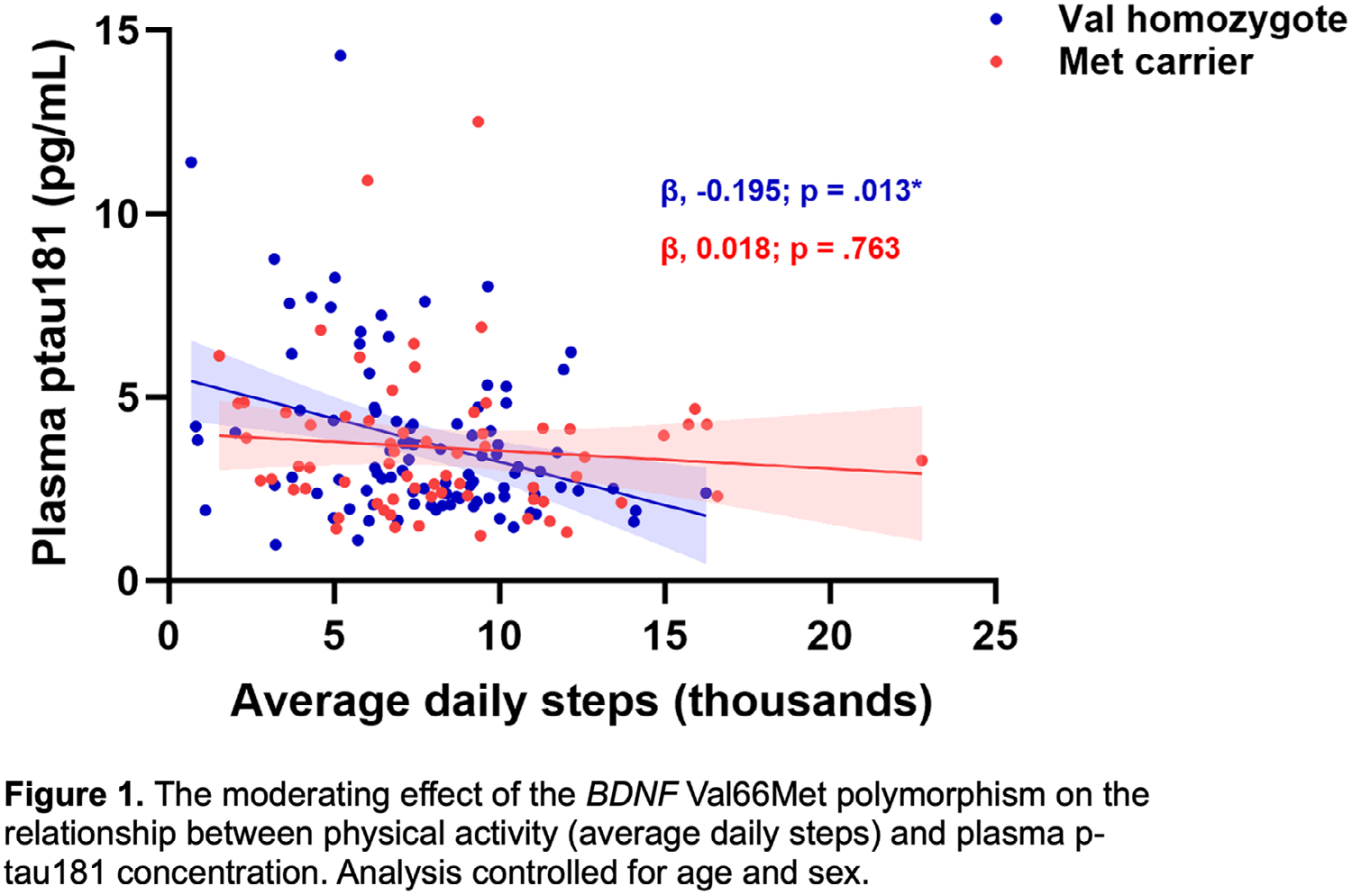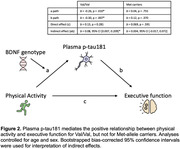# BDNF Val66Met polymorphism moderates the neuroprotective effects of physical activity in older adults

**DOI:** 10.1002/alz.092836

**Published:** 2025-01-09

**Authors:** Claire J. Cadwallader, David Luke Fischer, Anna M. VandeBunte, Coty Chen, Valentina E. Diaz, Shannon Y. Lee, Brandon Chan, Argentina Lario Lago, Julio C. Rojas, Joel H. Kramer, Emily W. Paolillo, Rowan Saloner, Kaitlin B. Casaletto

**Affiliations:** ^1^ Memory and Aging Center, UCSF Weill Institute for Neurosciences, University of California, San Francisco, San Francisco, CA USA; ^2^ Memory and Aging Center, UCSF Weill Institute for Neurosciences, University of California San Francisco, San Francisco, CA USA; ^3^ UCSF Alzheimer's Disease Research Center, San Francisco, CA USA; ^4^ Global Brain Health Institute, University of California San Francisco, San Francisco, CA USA

## Abstract

**Background:**

Physical activity (PA) is associated with increased release of brain derived neurotrophic factor (BDNF), a mechanism that may underlie protective effects of PA on cognitive and brain aging. The Met allele of the BDNF Val66Met single‐nucleotide polymorphism reduces activity‐dependent BDNF release and is associated with increased phosphorylated tau (p‐tau181) in dementia populations. We sought to determine whether BDNF Val66Met influences the effects of PA on plasma p‐tau181 and cognition in older adults without dementia.

**Method:**

123 older adults from the UCSF Memory and Aging Center completed blood draw assayed for p‐tau181 (Quanterix Simoa), BDNF Val66Met (rs6265) genotyping (Sequenom), 30‐days of Fitbit^TM^ actigraphy monitoring, and comprehensive neuropsychological evaluation (M_age_ = 73.93; SD_age_ = 8.88, 59% female, 42% Met‐allele carriers). Habitual PA levels were operationalized via average daily step count. Composite z‐scores were calculated for cognitive domains of memory and executive functioning. Linear regression was used to determine the moderating effect of BDNF genotype on the relationship between PA and plasma p‐tau181. Moderated mediation analyses subsequently examined the consequences of this moderation effect on cognitive outcomes.

**Result:**

Average PA, plasma p‐tau181, and cognitive z‐scores did not differ between Met‐allele carriers and Val/Val participants. BDNF genotype moderated the relationship between PA and plasma p‐tau181 (b = 0.18, p = 0.036), whereby higher PA was associated with lower plasma p‐tau181 concentrations in Val/Val participants only (Figure 1). In moderated mediation analyses examining cognitive outcomes, plasma p‐tau181 concentration mediated the relationship between PA and executive function for Val/Val participants only (index of mod‐med = ‐0.037, p = 0.031; Figure 2). Models examining memory did not reach statistical significance.

**Conclusion:**

Findings converge with prior studies highlighting BDNF release as a potential mechanism underlying the neuroprotective effects of physical activity, including lower AD pathological burden and better cognition. These results further elucidate sources of individual variation observed in the effects of PA on the brain in older adults. Future work should further examine the molecular pathways bridging the effects of the BDNF Val66Met polymorphism on PA‐related benefits for brain aging to guide personalized neurotrophic treatments in older adults.